# Pseudo-negative serum protein electrophoresis due to IgM aggregation in a patient with Waldenström macroglobulinemia: A case report

**DOI:** 10.1097/MD.0000000000050717

**Published:** 2026-09-11

**Authors:** Hanhan Xie, Enping He, Hui Quan, Shaocheng Zhang

**Affiliations:** a Department of Laboratory Medicine, The Second Affiliated Hospital of Chengdu Medical College (Nuclear Industry 416 Hospital), Chengdu, Sichuan Province, China.

**Keywords:** CXCR4, MYD88, refractory anemia, Waldenström macroglobulinemia, zanubrutinib

## Abstract

**Rationale:**

Waldenström macroglobulinemia (WM) is a type of immunoglobulin M (IgM)-secreting lymphoplasmacytic lymphoma primarily caused by the accumulation of clonal lymphocytes, lymphoplasmacytes, and plasma cells. The monoclonal IgM proteins secreted by these cells result in high blood viscosity.

**Patient concerns:**

A 79-year-old male presented with anemia lasting 10 months accompanied by edema in both lower limbs and testicles.

**Diagnoses:**

After a series of tests, including serological testing, serum protein electrophoresis, and genetic testing, the patient was ultimately diagnosed with WM.

**Interventions:**

The patient received a red blood cell suspension transfusion and targeted therapy with zanubrutinib.

**Outcomes:**

The patient’s dizziness and fatigue symptoms improved, no new blood blisters developed, and the anemia resolved.

**Lessons:**

This case highlights that clinicians should consider lymphomas such as WM in the differential diagnosis for elderly patients with refractory anemia that progressively worsens. Also, note that IgM aggregates in WM patients can cause false-negative electrophoresis results.

## 1. Introduction

Waldenström macroglobulinemia (WM) is a type of immunoglobulin M (IgM)-secreting lymphoplasmacytic lymphoma primarily caused by the accumulation of clonal lymphocytes, lymphoplasmacytes, and plasma cells. The monoclonal IgM proteins secreted by these cells result in high blood viscosity.^[1]^ Anemia is one of the primary symptoms of WM, occurring in over 80% of patients.^[2]^ In WM patients, the progressive growth of WM cells displaces normal erythropoiesis in the bone marrow, preventing red blood cell (RBC) production and causing anemia.^[3]^ When a significant increase in lymphoma cells is observed in the bone marrow of anemic patients, lymphoplasmacytic lymphoma should be considered. This report describes a case of WM presenting with refractory anemia (RA). The diagnostic process underscores the importance of integrating clinical symptoms and comprehensive test results when evaluating unexplained anemia.

## 2. Case analysis

**Figure 1.**
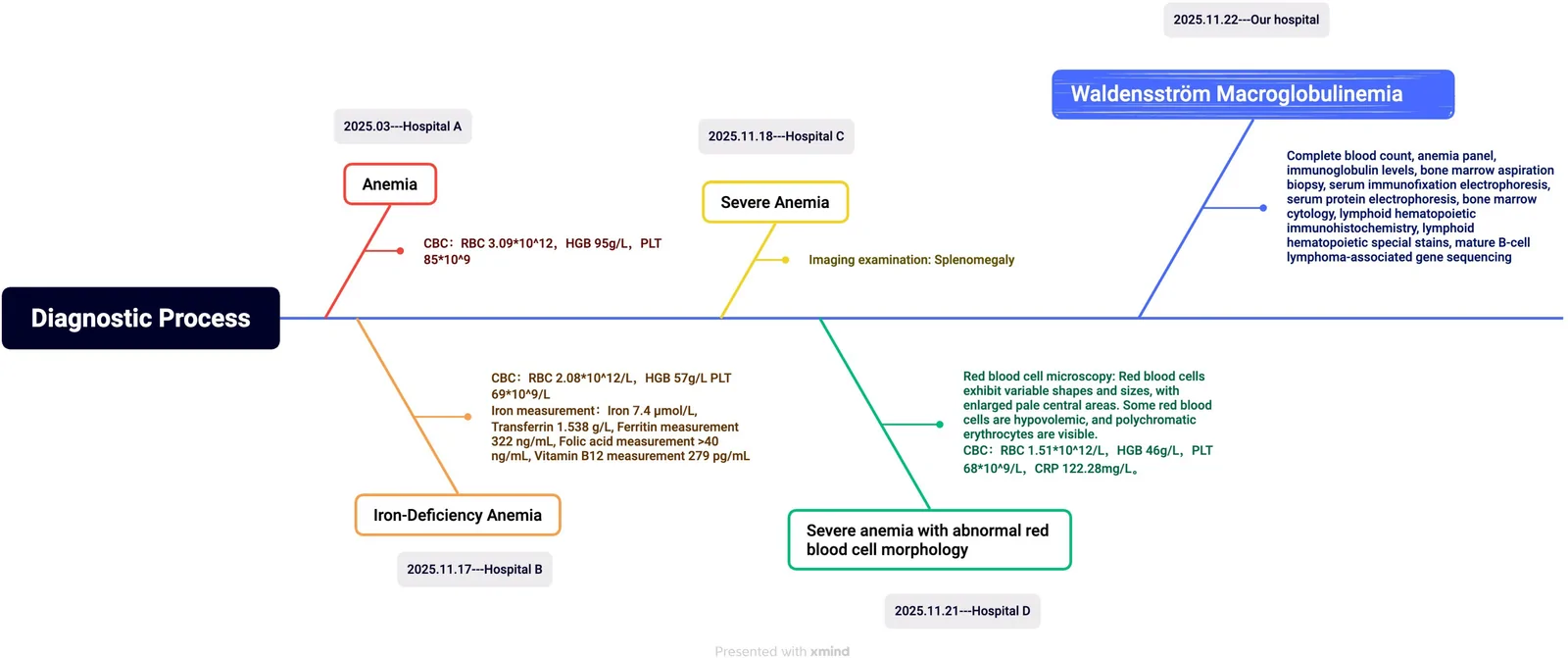
Diagnostic process of this case. CBC = complete blood count, CRP = C-reactive protein, HGB = hemoglobin, PLT = platelet, RBC = red blood cell.

**Figure 2.**
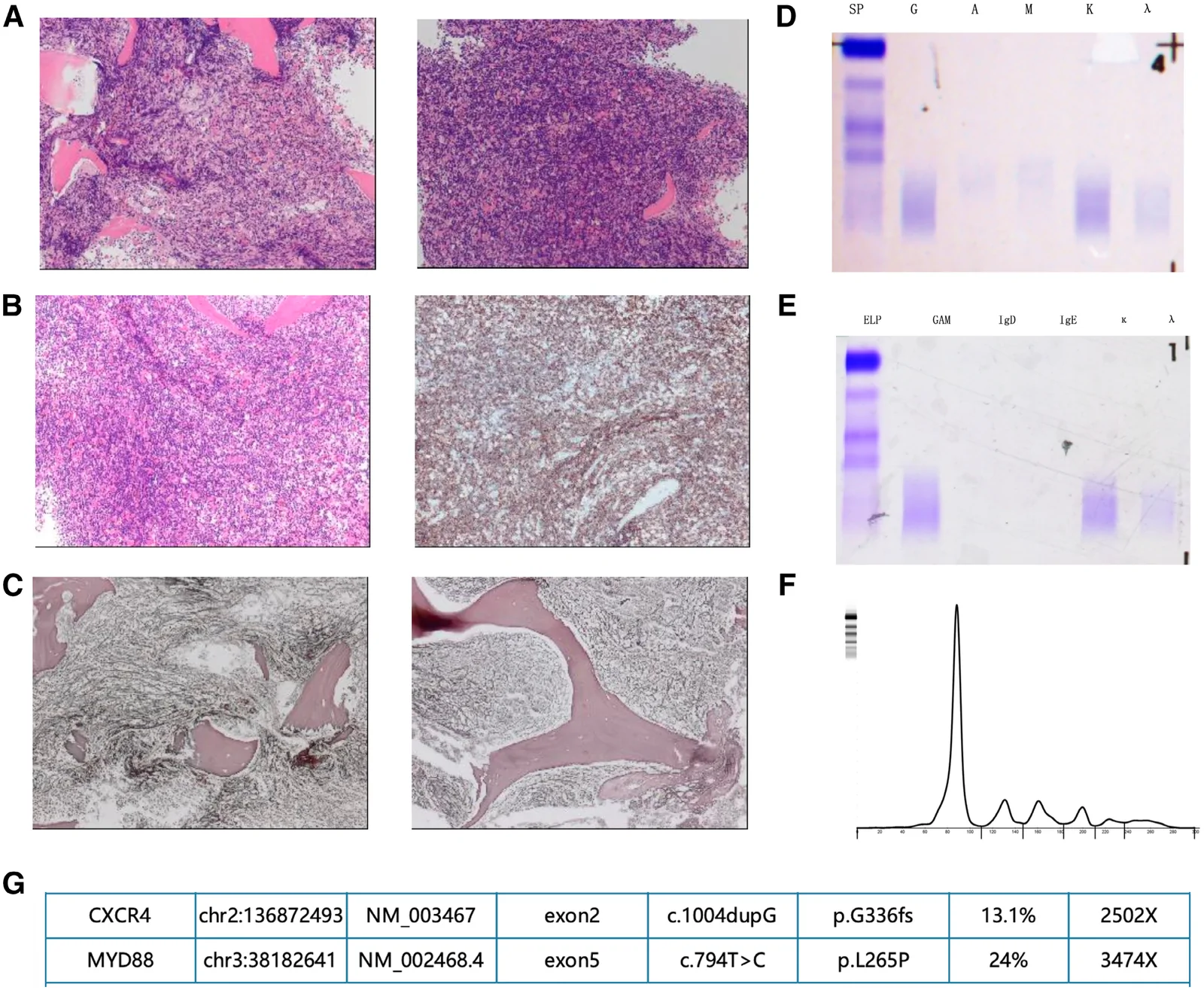
Various examination reports for the case. (a) Bone marrow tissue biopsy. (b) Lymphocyte immunohistochemistry. (c) Special staining. (d) Immunofixation electrophoresis (IgA + IgG + IgM). (e) Immunofixation electrophoresis (IgD + IgE). (f) Serum protein electrophoresis. (g) Genetic testing. *CXCR4* = CXC chemokine receptor 4, Ig = immunoglobulin, *MYD88* = myeloid differentiation primary response 88.

A 79-year-old male presented with anemia lasting 10 months, accompanied by edema in both lower limbs and testicles (Fig. 1). At hospital A, a complete blood count revealed decreased RBC count and reduced hemoglobin (HGB) concentration (RBCs: 3.09 × 10^12^/L and HGB: 95 g/L), leading to a diagnosis of “anemia.” Subsequently, his condition progressively worsened, prompting a visit to hospital B. Hospital B’s complete blood count revealed further deterioration of anemia parameters (RBC: 2.08 × 10^12^/L and HGB: 57 g/L) with concomitant iron deficiency, leading to a diagnosis of “iron deficiency anemia (IDA).” Computed tomography at hospital C revealed splenomegaly, and the patient’s blood exhibited high viscosity. Peripheral blood morphology examination at hospital D showed abnormal RBC morphology: variable size, enlarged pale central areas, some microcytic cells, and polychromatic erythrocytes. Immunohistochemistry at our hospital demonstrated k-light chain restriction pattern, with immunophenotyping showing cluster of differentiation 20+ and kappa+ profiles (Fig. 2A and B). Bone marrow biopsy revealed lymphoplasmacytic infiltration (80%) (Fig. 2A). Special staining for lymphoproliferative disorders reveals a reticular pattern characterized by diffuse reticular fibrous proliferation with focal collagen deposition (myelofibrosis grade 2). (Fig. 2C). A suspected precipitated band was observed in the κ lane of serum immunofixation electrophoresis, indicating a monoclonal immunoglobulin of the kappa free light chain type (Fig. 2D and E). Because of the influence of IgM aggregates, no M protein peak was observed in the serum protein electrophoresis (SPEP) profile (Fig. 2F). Genetic testing identified CXC chemokine receptor 4 (*CXCR4*) gene p.G336fs mutation and myeloid differentiation primary response 88 (*MYD88*) gene p.L265P mutation; variant abundances were 14.1% and 24% (Fig. 2G), respectively. Serological confirmation demonstrated IgM monoclonal gammopathy (IgM immunoglobulin 20 g/L↑). The comprehensive diagnosis is WM.

The patient had previously received oral iron supplementation for diagnosed IDA – IDA is a type of microcytic hypochromic anemia caused by insufficient iron levels in the body, which leads to reduced HGB synthesis,^[4]^ but the anemia did not improve. After transfer to our hospital, clinical symptoms were assessed, and based on the Chinese Guidelines for the Diagnosis and Treatment of Waldenström Macroglobulinemia (2025 Edition), the patient was administered single-agent targeted therapy with zanubrutinib. Four days after his initial admission to our hospital, he began receiving blood transfusions. During his 2 hospital stays, he received a total of 12.5 units of RBC suspension. Upon his second admission, he was diagnosed with WM and began targeted therapy with zanubrutinib at a dose of 80 mg, taken once in the morning and once in the evening, with a 12-hour interval between doses. The patient has been discharged and is continuing to take medication. The patient’s dizziness and fatigue symptoms improved, no new blood blisters developed, and the anemia resolved (Table 1).

**Table 1:** Comparison of complete blood count results before and after

Clinical laboratory test	2025.11.26	2026.01.07	2026.01.11	Reference range
WBC (10^9^/L)	2.02	4.14	3.39	3.5–9.5
RBC (10^12^/L)	0.83	1.11	2.05	4.3–5.8
HGB (g/L)	21	31	58	130.00–175.00
HCT (%)	7.9	9.4	19.4	40.00–50.00
MCV (fL)	95.2	84.7	94.6	82.00–100.00
MCH (pg)	25.3	27.9	28.3	27.00–34.00
MCHC (g/L)	266	330	299	316.00–354.00
PLT (×10^9^/L)	33	39	37	125.00–350.00

HCT = hematocrit, HGB = hemoglobin, MCH = mean corpuscular hemoglobin, MCHC = mean corpuscular hemoglobin concentration, MCV = mean corpuscular volume, PLT = platelets, RBC = red blood cells, WBC = white blood cells.

## 3. Discussion

This case demonstrates that in patients with RA accompanied by hyperviscosity symptoms, a high index of suspicion should be held for lymphoplasmacytic proliferative disorders such as WM. Diagnostic confirmation requires serological testing, bone marrow examination, and genetic sequencing. Both RA and WM can present with anemia, but the differential diagnosis between the 2 must be based on the nature of the disease. RA, as a subtype of myelodysplastic syndrome, is characterized by pathological hematopoiesis in clonal hematopoietic stem cells in the bone marrow, manifesting as monolineage or multilineage developmental abnormalities and ineffective hematopoiesis.^[5]^ In contrast, WM is an IgM-associated lymphoplasmacytic lymphoma characterized by clonal lymphoplasmacytic infiltration of the bone marrow, accompanied by monoclonal IgM protein in the serum. Although patients with WM also frequently develop anemia because of factors such as bone marrow infiltration and inflammation, their normocytic normochromic anemia is often accompanied by hyperviscosity syndrome, hepatosplenomegaly, or lymphadenopathy. Therefore, in patients with RA of unknown origin who also present with an elevated erythrocyte sedimentation rate or elevated serum globulin levels, WM should be considered. A bone marrow biopsy, SPEP, and immunofixation electrophoresis should be performed for differential diagnosis; testing for the *MYD88 L265P* mutation can help further confirm the diagnosis of WM.

WM typically presents insidiously and slowly, often without discomfort in the early stages, with anemia frequently manifesting during disease progression. In this case, anemia presented as the initial symptom. The diagnostic process was somewhat challenging due to the wide variety of anemia-related conditions and the patient’s noncompliance. Initially, the patient’s classic anemia appearance, markedly reduced hematologic parameters, iron deficiency, and altered RBC morphology led to a misdiagnosis of “IDA.” During treatment, the presence of a hypercoagulable state – a key hallmark of WM – shifted the diagnostic focus from common anemia-related disorders toward lymphoproliferative diseases. Subsequent serological, bone marrow, lymphocyte immunohistochemical, and high-throughput genetic testing confirmed WA. Serological analysis revealed no M-protein band in the serum electrophoresis pattern, while blood IgM levels were elevated (20 g/L), potentially because of limitations of the detection method. The M-protein detection employed capillary electrophoresis. A plausible explanation for the absence of an M-protein peak is that large IgM aggregates may have obstructed the passage of monoclonal proteins through the narrow capillary tubes (inner diameter < 100 μm), leading to a negative electrophoretic pattern. In such cases, clinical context must be considered comprehensively to avoid diagnostic delays because of the absence of a protein peak.

Genetic testing in this case revealed *MYD88 L265P* and *CXCR4 G336fs* mutations, providing strong evidence for a diagnosis of WM. These 2 somatic mutations exhibit high mutation rates in WM patients, and their presence correlates with the efficacy of targeted therapies.^[6,7]^ Studies indicate that the *MYD88 L265P* mutation occurs in over 90% of WM cases.^[6]^ Under normal conditions, the *MYD88* gene regulates B-cell differentiation and maturation, aiding in the elimination of self-reactive clonal populations.^[8]^ The *MYD88 L265P* mutation causes *MYD88* to spontaneously form homodimers and recruit downstream interleukin-1 receptor-associated kinase 1 and interleukin-1 receptor-associated kinase 4, leading to uncontrolled formation of the MYD88/interleukin-1 receptor-associated kinase complex. This ultimately activates nuclear factor kappa-light-chain-enhancer of activated B cells, thereby promoting malignant proliferation of lymphoplasmacytic cells.^[9,10]^ In most individuals with *MYD88* mutations, *CXCR4* mutations frequently co-occur. Approximately 29% of patients exhibit this gene mutation, and 53% of patients with high viscosity present *CXCR4* mutations.^[11]^ G336fs is one of the common mutation types. *CXCR4* mutations correlate with clinical manifestations (e.g., lymphadenopathy, serum IgM levels, and bone marrow tumor burden) and the invasive capacity of lymphoplasmacytic cells (e.g., bone marrow adhesion and systemic organ invasion).^[12]^

## 4. Conclusion

This case highlights that clinicians should consider lymphomas such as WM in the differential diagnosis for elderly patients with RA that progressively worsens. Serological, cytological, and bone marrow examinations are essential diagnostic tools. When necessary, electrophoresis, immunohistochemical staining, and genetic testing should be employed to refine the diagnostic process and confirm the disease diagnosis. At the same time, in cases where serum IgM levels are extremely high but SPEP fails to show a monoclonal (M) protein peak, one should also consider the possibility of a false-negative result due to methodological limitations, which could mask severe hypergammaglobulinemia.

## Author contributions

**Conceptualization:** Hanhan Xie, Shaocheng Zhang.

**Data curation:** Enping He.

**Formal analysis:** Hui Quan.

**Investigation:** Hui Quan.

**Funding acquisition:** Shaocheng Zhang.

**Writing – original draft:** Hanhan Xie.

**Writing – review & editing:** Hanhan Xie, Shaocheng Zhang.
